# A call for SASOP to review their position on the care of ‘transgender and non-binary youth’

**DOI:** 10.4102/sajpsychiatry.v31i0.2381

**Published:** 2025-04-11

**Authors:** Allan Donkin, Reitze Rodseth, Janet Giddy

**Affiliations:** 1 First Do No Harm Southern Africa, Cape Town, South Africa; 2 Department of Anaesthesiology/Research Methodology, Faculty of Medicine, University of KwaZulu-Natal, Pietermaritzburg, South Africa

## Background

The South African Society of Psychiatrists (SASOP) aims to promote, maintain and honour the discipline of psychiatry as a medical speciality and to serve the community by providing information and guidance for those in need of professional help. In March 2024, SASOP published a position statement: *SASOP CAPSIG Position Statement on the Care of Transgender and Non-binary Youth,*^1^ recommending a ‘gender affirming care (GAC)’ approach for minors, including medical interventions. The CAPSIG is the Child and Adolescent Psychiatry Special Interest Group, within SASOP.

‘Gender affirming care’ includes:^2,3^ (1) *social transition* at any age, including young children; (2) the off-label use of *drugs to block puberty* in adolescents and young adults; (3) the off-label use of *cross-sex hormones*; and (4) *body altering surgery*, such as mastectomy and penis amputations.

The SASOP position statement uses the World Professional Association of Transgender Health (WPATH) Standards of Care Version 8 (SOC8)^2^ and the Southern African HIV Clinicians Society (SAHCS) Gender Affirming Healthcare Guideline (GAHG)^3^ as the foundation of its GAC recommendations.

On 10 April 2024 the Cass Review was released by the National Health Service in the UK.^4^ Led by paediatrician Dr Hilary Cass, the report is the culmination of a 4-year investigation into how the National Health Service (NHS) provided care to children and young people with gender dysphoria. The report, accompanied by nine studies, eight of which were systematic reviews, marked a drastic departure from the GAC approach to the treatment of young people with gender-related distress in the UK. The Cass Review concludes that ‘gender affirming’ medical treatments are based on ‘wholly inadequate evidence’.^4^ Recent critiques of the Cass review, such as those by the Yale Integrity Project, have in turn been criticised because of author conflict of interest and reliance on poor quality evidence to support GAC.^5,6^

First Do No Harm SA (FDNHSA) is a voluntary association of Southern African professionals advocating for evidence-based care of children and young people with gender distress or incongruence. On 18 March 2024 FDNHSA engaged with SASOP, to express concerns regarding SASOP’s position statement and its implications for children. On 23 April the SASOP Board responded formally to the concerns, stating ‘(we) remain in support of the position statement, based on the consensus discussion of our members’ (Porter A 2024, [*SASOP Board Secretary*], personal communication, April 23).

In this letter, we explain the concerns FDNHSA raised and again call on the SASOP Board to revise their position statement.

## Framing of the position statement

A position statement is a document outlining the stance of a professional body on a specified area. The SASOP statement has in the title ‘… care of transgender and non-binary youth (TGBN)’, and the first paragraph of the statement notes:

‘Some people who are transgender experience gender dysphoria (DSM 5) which refers to psychological distress that results from this [*gender*] incongruence.’^1^

This inappropriately frames all children with gender dysphoria as being a subset of those who are ‘transgender’. No definition is given for ‘transgender and non-binary youth’ (‘TGNB’), which do not appear in both the Diagnostic and Statistical Manual of Mental Disorders, Fifth Edition, Text Revision (DSM-5-TR) and the International Classification of Diseases, 11th Revision (ICD11) as diagnostic categories nor in Standards of Care Version 8 (SOC8) and the SAHCS GAHG, which use ‘transgender and gender diverse (TGD)’.

While there has been a surge in the number of children and adolescents presenting to clinicians with gender dysphoria there are many possible causes for this dysphoria, including autism, depression, anxiety, obsessive compulsive disorder, history of trauma or abuse, family disruption and social contagion.

Until the recent adoption of the GAC pathway, most children found that their symptoms resolved as they passed through puberty and that their care did not require social transition or hormonal therapy. The SASOP position statement ignores this. Having gender distress does not mean that a child is ‘transgender’, the definition of which is not given. For SASOP, as a professional medical body that aims to serve the community by providing information and guidance, this is a significant oversight.

## Rigour of development

Position statements must be developed in a rigorous, scientific and professional manner, and reflect the highest quality research evidence available. This is particularly important when the long-term health of children and adolescents is at stake. Global best practice is to use a tool such as The Appraisal of Guidelines for Research & Evaluation (AGREE) Instrument.^7^ The SASOP position statement was not developed through an AGREE II or similar process, and is not based on the highest quality evidence, but rather:

‘A team of child and adolescent psychiatrists was tasked with reviewing the literature. They reviewed local and international guidelines as well as more recent publications such as the CASS interim report.’^1^

Even while referencing the Cass review, which concluded that ‘gender affirming’ medical treatments are based on ‘wholly inadequate evidence’, the SASOP position statement goes on to state:

‘There is significant evidence to support the improvement of dysphoria and the mental health of transgender individuals when they are supported in their gender identities. It has been well established that access to gender affirming health care is both safe and can contribute to improvement in a number of measurable outcomes.’^1^

Regarding the (off label) use of puberty suppressing hormones (PSH) for GAC, it should be noticed that a rigorous process of consulting multiple high-quality systematic reviews has led to the use of PSH being restricted to research settings in countries such as the UK:

‘NHS England recommends that access to PSH for children and young people with gender incongruence [*or*] dysphoria should only be available as part of research.’^8^

Further afield, in Finland, following a systematic review of evidence, the Council for Choices in Health Care (COHERE) emphasised the need for an adolescent to first complete their developmental tasks and for other psychiatric illnesses to have stabilised or improved before a gender identity assessment will be considered. The Finnish statement reads:

‘If an adolescent experiencing gender anxiety has had or has concomitant psychiatric symptoms requiring specialized care, in case the need for it continues after the psychiatric symptoms have yielded and the adolescent development tasks’ progress has normalised, it is possible to consider gender identity examinations.’^9^

The two guidelines promoted by the SASOP position statement (SOC8, SAHCS GAHG) underwent formal and detailed systematic review, using AGREE II, as part of the Cass review process. The first review was of guideline quality. The second review was on the quality of the recommendations made in the guidelines. Both of them scored poorly on guideline quality, as well as the quality of their recommendations.^10,11^

A strength of the SASOP position statement is that it recommended the review of guidelines in the light of new evidence.

## Reliance on selected research articles and position statements

Engaging as broadly as possible with good quality research evidence related to a topic is essential in developing a position statement. As highlighted, the SASOP position statement ignored the Cass Review, and other guidelines issued by national health authorities. No statement or claim in the position statement is specifically linked to the citations in ‘References’ and ‘Additional References’. The position statement draws from publications that have been extensively criticised for their poor quality, such as those by Chen et al. and Tordoff et al. Chen et al.’s article has been criticised for its failure to report on all parameters studied, the extremely small improvements reported in the few parameters that were reported on, the authors refusal to release raw data for closer inspection, a worryingly high suicide rate in the cohort treated with hormones, no control group, selection bias, confounding factors and the disregard for the honeymoon period of elevated mood experienced with the initiation of cross-sex hormones.^12^ Tordoff et al.’s article has been criticised^13^ for the 80% drop out rate in the control group, selection bias, refusal of the authors to share their data and problems with their statistical methods.

In the position statement reference list are the two guidelines endorsed by SASOP – WPATH SOC8,^2^ and SAHCS GAHG,^3^ with no evidence or reasoning provided for this endorsement. However, support for the position statement is claimed through various United States based medical organisations, including the AACAP, AAP, APA, AMA, ACOG and the American Association of Clinical Endocrinology all of which support ‘gender affirming’ care. None of these organisations has used global best practice for developing GAC recommendations nor have they drawn their conclusions from systematic reviews. Instead, they have been shown to be involved in circular referencing and mutual endorsements. A detailed account of this was presented in the one of the Cass systematic reviews.^10^

The SASOP position statement itself gives a clear example of circular referencing where it states that the Professional Association of Transgender Health (PATHSA), an affiliate of WPATH, endorses the SAHCS GACG. Most of the authors of the SAHCS GACG are members of PATHSA. The PATHSA therefore endorses the guideline it authored.

Additional concern was raised with the release in March 2024 of the ‘WPATH Files: Pseudoscientific surgical and hormonal experiments on children, adolescents, and vulnerable adults’.^14^ This record of internal WPATH communications makes public WPATH’s own concerns about the inadequate evidence base for their recommendations and about ‘GAC’ informed consent processes. Also, documents unsealed in the US Alabama court case involving WPATH, revealed that WPATH suppressed their own systematic reviews commissioned to the Johns Hopkins University Evidence-based Practice Centre, and exposed political interference in the process of their SOC8 development.^15^

At the time, The Guardian newspaper reported on the nature of WPATH:

‘WPATH describes itself as an “interdisciplinary professional and educational organization devoted to transgender health.” Most significantly, it produces standards of care (SOC) which, it claims, articulate “professional consensus” about how best to help people with gender dysphoria. Despite its grand title, WPATH is neither solely a professional body – a significant proportion of its membership are activists – nor does it represent the “world” view on how to care for this group of people.’^16^

World Professional Association of Transgender Health is the organisation endorsed in the SASOP position statement.

## Informed consent

Informed consent, as it relates to the ability of children and adolescents with developing brains to be able to competently agree to GAC, is a very serious matter, involving decisions about medical and surgical interventions with long-term and permanent effects. The SAHCS GAHG states:

‘In South Africa, a child who is over 12 years of age, and meets these stipulated criteria, may independently give informed consent to both psychosocial and medical assessment/s and intervention for GAHC without legally requiring the support or consent of their parents [*or*] legal guardians.’^3^

Specifically for puberty blockade and cross-sex hormones the SAHCS GAHG states:

‘In South Africa, an adolescent aged 12 years and older, deemed competent and of sufficient capacity, can give informed consent (IC) to this treatment (puberty suppression with GnRHa). This IC principle would also apply in cases where the introduction of gender-affirming HT (hormone treatment) in later adolescence is considered appropriate and necessary for the adolescent.’^3^

World Professional Association of Transgender Health members themselves, as exposed in the WPATH Files, acknowledge that adolescents cannot make informed decisions about interventions that cause sterility and anorgasmia.^14^

In the position statement, SASOP is silent on this critical issue of informed consent, and in the silence, endorses the WPATH and SAHCS GAHG informed consent approach.

## Alternatives to gender affirming care

The position statement makes no mention of the alternative treatment strategies for gender distress such as neutral exploratory psychotherapy, attention to family and social circumstances and management of co-existing psychiatric conditions. All have been well-described for the management of children with gender distress.^4^

## Risks and harm of gender affirming care

There is a growing evidence-base showing harms related to ‘GAC’. In addition to the iatrogenic harms inherent in the use of cross-sex hormones (sterility and anorgasmia), there are serious concerns about the adverse neurodevelopmental effects of using puberty blockers in young patients.^17^

Rigorous long-term patient follow-up of GAC to assess safety and harm has not been performed. Even in the Dutch gender clinic, when they reported on 20 years of clinic experience,^18^ the median follow-up per patient was only 4.6 years from the first intake appointment. For a quarter of cases the follow-up was less than 3 years.^19^

A further worrying component of the GAC approach is the ‘social transition’ of children. In 2022, Dr Kristina Olsen published a study where she reported that 5 years after socially transitioning children who ‘identified as transgender’, only 7% retransitioned to live as their original biological sex.^20^ This is in stark contrast to the historically well-known pattern that most children presenting with a cross-sex identity or gender dysphoria would not persist with these beyond puberty when not socially transitioned.^21^

The SASOP position statement makes no mention of a ‘child’s right to an open future’. This ethical principle states that children have the right to have their future life choices kept open until they reach adulthood, so they can exercise them in the future as autonomous adults.^22^

There is a growing number of detransitioners in countries where GAC has been practiced over the last decade. This has been accompanied by a number of high profile court cases being brought against doctors, where serious and permanent harm has occurred. In one of many such cases, Clementine Breen who had her breasts removed at the age of 14, is suing her doctor, the paediatrician Dr Olson-Kennedy, who is a WPATH member who advocated for mastectomy as treatment for gender dysphoria for her as an adolescent girl.^23^

The SASOP position statement makes no mention of harms or medico-legal risks.

## Conclusion

Medical societies need to provide sound guidance to their members and to the public and have an ethical responsibility to comprehensively and objectively review the evidence supporting a position statement. The SASOP position statement on ‘the care of transgender and non-binary youth’ has serious shortcomings and does not properly engage with the wealth of new literature on the subject. South African children and adolescents with gender dysphoria or incongruence need to be protected from the extreme medical and surgical interventions packaged as ‘GAC’ and promoted by WPATH, the SAHCS and in the SASOP position statement. The SASOP can play a significant role in putting safeguards for children in place, and it has a responsibility to do so.

Apart from re-evaluation based on new evidence, AGREE II can be used by professional organisations to help decide which guidelines could be recommended for use in practice or to inform policy decisions, in this case the SASOP position statement. As much of the evidence is new and has led to changes in guidelines in several countries, we recommend this as a way forward for SASOP.

## References

[1] SASOP. SASOP CAPSIG position statement on the care of transgender and non-binary youth South Africa: South African Society of Psychiatrists; 2024 [cited 2025 Feb]. [Position Statement]. Available from: https://www.sasop.co.za/_files/ugd/cc5d8c_df929cb80453462487b93af53abb69b7.pdf 10.4102/sajpsychiatry.v31i0.2381 PMC12067532 40357173

[2] Coleman E, Radix AE, Bouman WP, et al. Standards of care for the health of transgender and gender diverse people, version 8 [homepage on the Internet]. Int J Transgen Health. 2022;23(Supp1):S1–S259. 10.1080/26895269.2022.2125695 PMC9553112 36238954

[3] Tomson A, McLachlan C, Wattrus C, et al. Southern African HIV Clinicians Society gender-affirming healthcare guideline for South Africa – Expanded version [homepage on the Internet]. 2021 [cited 2025 Feb]. Available from: https://sahivsoc.org/Subheader/Index/sahcs-guidelines 10.4102/sajhivmed.v22i1.1299 PMC8517808 34691772

[4] Cass H. Independent review of gender identity services for children and young people: Final report. London: NHS England; 2024.

[5] Cheung CR, Abbruzzese E, Lockhart E, Maconochie IK, Kingdon CC. Gender medicine and the Cass Review: Why medicine and the law make poor bedfellows. Arch Dis Childhood. 2025;110(4):251–255. 39401844 10.1136/archdischild-2024-327994 PMC12013558

[6] McDeavitt K. Citation issues in the american academy of pediatrics policy statement on transgender and gender-diverse children and adolescents (Rafferty, 2018). Archives of Sexual Behavior [Internet]. 2025 [cited 2025 Feb 05]. Available from: https://link.springer.com/article/10.1007/s10508-025-03106-5 10.1007/s10508-025-03106-5 PMC12011888 39907844

[7] Brouwers MC, Kho ME, Browman GP, et al. AGREE II: Advancing guideline development, reporting and evaluation in health care. CMAJ. 2010;182(18):E839–E842. 10.1503/cmaj.090449 20603348 PMC3001530

[8] Miller R, Lund K, Scott M. Interim clinical policy: Puberty suppressing hormones (PSH) for children and adolescents who have gender incongruence or dysphoria. London: NHS; 2024.

[9] COHERE. Recommendation of the Council for Choices in Health Care in Finland: Medical treatment methods for dysphoria related to gender variance in minors Finland: PALKO/COHERE [homepage on the Internet]. 2020 [cited 2025 Feb]. [Unofficial Translation]. Available from: https://segm.org/sites/default/files/Finnish_Guidelines_2020_Minors_Unofficial%20Translation.pdf

[10] Taylor J, Hall R, Heathcote C, Hewitt CE, Langton T, Fraser L. Clinical guidelines for children and adolescents experiencing gender dysphoria or incongruence: A systematic review of guideline quality (part 1). Arch Dis Child. 2024;109(Suppl. 2):s65–s72. 10.1136/archdischild-2023-326499 38594049

[11] Taylor J, Hall R, Heathcote C, Hewitt CE, Langton T, Fraser L. Clinical guidelines for children and adolescents experiencing gender dysphoria or incongruence: A systematic review of recommendations (part 2). Arch Dis Child. 2024;109(2):s73–s82. 10.1136/archdischild-2023-326500 38594048

[12] Singal J. On scientific transparency, researcher degrees of freedom, and that NEJM study on youth gender medicine [homepage on the Internet]. Substack; 2023 [cited 2025 Feb]. Available from: https://substack.com/@jessesingal

[13] Singal J. The new, highly touted study on hormones for transgender teens doesn’t really tell us much of anything: Substack [homepage on the Internet]; 2023 [cited 2025 Feb]. Available from: https://substack.com/@jessesingal

[14] Hughes M. The WPATH files: Pseudoscientific surgical and hormonal experiments on children, adolescents, and vulnerable adults. Berkeley, CA: Environmental Progress; 2024.

[15] Marshall S. Brief of Alabama as Amicus Curiae supporting state respondents. USA v. Skrmetti et al., Alabama: SCOTUS; 2024, p. 23–447.

[16] Barnes H. Why disturbing leaks from the US gender group WPATH ring alarm bells in the NHS. The Guardian [Internet]. 2024 [cited 2025 Feb]. Available from: https://www.theguardian.com/commentisfree/2024/mar/09/disturbing-leaks-from-us-gender-group-wpath-ring-alarm-bells-in-nhs

[17] Baxendale S. The Impact of Suppressing Puberty on Neuropsychological Function. Authorea. 2024. 10.22541/au.170446841.14546991/v3 38334046

[18] Van der Loos M, Klink D, Hannema S, et al. Children and adolescents in the Amsterdam Cohort of Gender Dysphoria: Trends in diagnostic- and treatment trajectories during the first 20 years of the Dutch Protocol. J Sex Med. 2023;20(3):398–409. 10.1093/jsxmed/qdac029 36763938

[19] Society for Evidence-based Gender Medicine. New “20-year” Study from Amsterdam’s VUmc youth gender clinic: A critical analysis [homepage on the Internet]. 2023 [cited 2025 Feb]. Available from: https://segm.org/20-years-of-the-Dutch-Protocol-critical-analysis

[20] Olson KR, Durwood L, Horton R, Gallagher NM, Devor A. Gender identity 5 years after social transition. Pediatrics. 2022;150(2):e2021056082. 10.1542/peds.2021-056082 35505568 PMC9936352

[21] Ristori J, Steensma TD. Gender dysphoria in childhood. Int Rev Psychiatry. 2016;28(1):13–20. 10.3109/09540261.2015.1115754 26754056

[22] Jorgensen SC, Athéa N, Masson C. Puberty suppression for pediatric gender dysphoria and the child’s right to an open future. Arch Sex Behav. 2024;53(5):1941–1956. 10.1007/s10508-024-02850-4 38565790 PMC11106199

[23] Schwanemann K. UCLA student sues California doctors, says she was ‘fast-tracked’ into transgender surgery. NBC News [homepage on the Internet]. 2024 [2025 Feb]. Available from: https://www.nbcnews.com/nbc-out/out-news/ucla-student-sues-california-doctors-says-was-fast-tracked-transgender-rcna183815

